# Preoperative prediction of sonic hedgehog and group 4 molecular subtypes of pediatric medulloblastoma based on radiomics of multiparametric MRI combined with clinical parameters

**DOI:** 10.3389/fnins.2023.1157858

**Published:** 2023-04-11

**Authors:** Yuanlin Wang, Longlun Wang, Bin Qin, Xihong Hu, Wenjiao Xiao, Zanyong Tong, Shuang Li, Yang Jing, Lusheng Li, Yuting Zhang

**Affiliations:** ^1^Ministry of Education Key Laboratory of Child Development and Disorders, Chongqing Key Laboratory of Pediatrics, National Clinical Research Center for Child Health and Disorders, China International Science and Technology Cooperation Base of Child Development and Critical Disorders, Department of Radiology, Children’s Hospital of Chongqing Medical University, Chongqing, China; ^2^Department of Radiology, Children’s Hospital of Fudan University, Shanghai, China; ^3^Huiying Medical Technology Co., Ltd., Beijing, China; ^4^Ministry of Education Key Laboratory of Child Development and Disorders, Chongqing Key Laboratory of Pediatrics, National Clinical Research Center for Child Health and Disorders, China International Science and Technology Cooperation Base of Child Development and Critical Disorders, Department of Neurosurgery, Children’s Hospital of Chongqing Medical University, Chongqing, China

**Keywords:** medulloblastoma, radiomics, molecular subtypes, machine learning, prediction models

## Abstract

**Purpose:**

To construct a machine learning model based on radiomics of multiparametric magnetic resonance imaging (MRI) combined with clinical parameters for predicting Sonic Hedgehog (SHH) and Group 4 (G4) molecular subtypes of pediatric medulloblastoma (MB).

**Methods:**

The preoperative MRI images and clinical data of 95 patients with MB were retrospectively analyzed, including 47 cases of SHH subtype and 48 cases of G4 subtype. Radiomic features were extracted from T1-weighted imaging (T1), contrast-enhanced T1 weighted imaging (T1c), T2-weighted imaging (T2), T2 fluid-attenuated inversion recovery imaging (T2FLAIR), and apparent diffusion coefficient (ADC) maps, using variance thresholding, SelectKBest, and Least Absolute Shrinkage and Selection Operator (LASSO) regression algorithms. The optimal features were filtered using LASSO regression, and a logistic regression (LR) algorithm was used to build a machine learning model. The receiver operator characteristic (ROC) curve was plotted to evaluate the prediction accuracy, and verified by its calibration, decision and nomogram. The Delong test was used to compare the differences between different models.

**Results:**

A total of 17 optimal features, with non-redundancy and high correlation, were selected from 7,045 radiomics features, and used to build an LR model. The model showed a classification accuracy with an under the curve (AUC) of 0.960 (95% CI: 0.871−1.000) in the training cohort and 0.751 (95% CI: 0.587−0.915) in the testing cohort, respectively. The location of the tumor, pathological type, and hydrocephalus status of the two subtypes of patients differed significantly (*p* < 0.05). When combining radiomics features and clinical parameters to construct the combined prediction model, the AUC improved to 0.965 (95% CI: 0.898−1.000) in the training cohort and 0.849 (95% CI: 0.695−1.000) in the testing cohort, respectively. There was a significant difference in the prediction accuracy, as measured by AUC, between the testing cohorts of the two prediction models, which was confirmed by Delong’s test (*p* = 0.0144). Decision curves and nomogram further validate that the combined model can achieve net benefits in clinical work.

**Conclusion:**

The combined prediction model, constructed based on radiomics of multiparametric MRI and clinical parameters can potentially provide a non-invasive clinical approach to predict SHH and G4 molecular subtypes of MB preoperatively.

## 1. Introduction

Medulloblastoma (MB) is one of the most common malignant brain tumors, and accounts for 15−20% of central nervous system tumors in children and 40% of tumors in the posterior cranial fossa (Kumar et al., 2015; Massimino et al., 2016; Northcott et al., 2019). Prior to the emergence of molecular diagnostics, MB was classified histologically into subtypes including classic, extensive nodularity, desmoplastic or nodular, and large cell or anaplastic. However, recent studies have found that histopathological classification does not provide better prediction for the prognosis of patients and guidance of clinical treatment (Louis et al., 2016; Massimino et al., 2016).

With the development of molecular diagnostic techniques, the 2016 World Health Organization (WHO) classification of central nervous system tumors classified MB into four molecular subtypes, including wingless (WNT), sonic hedgehog (SHH, TP53 mutant, or wild type), Group 3 (G3), and Group 4 (G4) (Louis et al., 2016). Different molecular subtypes have different molecular mechanisms, clinical characteristics, and prognosis (Eid and Heabah, 2021; Fang et al., 2022). The WNT-activated type, which accounts for approximately 10% of MB, with a 1:1 male and female incidence ratio, originates in the rhombomere lip and dorsal brainstem of older children, has the best clinical outcomes, and is usually accompanied by an exon 3 activating mutation in *CTNNB1* and Chromosome 6 monomers (Ramaswamy et al., 2016; Colafati et al., 2018). The SHH-activated type, which accounts for approximately 30% of MB, originating from cerebellar granule cells, has a moderate prognosis, and the common molecular variants are *TP53*, *PTCH1*, *SUFU*, *SMO*, and other genes mutations (Colafati et al., 2018; Waszak et al., 2018; Hovestadt et al., 2019). In the non-WNT/SHH-activated type (G3 and G4), which accounts for approximately 25 and 35% of MB, respectively, the main common molecular variants are frequent *MYC*, *MYCN*, and, *OTX2* amplification. Patients in the G4 group with i17q or chromosome 11 deletion have a better prognosis compared to G3, but the prognosis of G4 is significantly worse compared to SHH or WNT-activated types (Zhao et al., 2016; Archer et al., 2017).

The published methods of molecular classification are invasive, relying mostly on gene expression and methylation analyses. In recent years, rapid advances in radiomics and machine learning techniques have made it possible to preoperatively predict MB molecular subtypes non-invasively. Radiomics is a quantitative analysis method of standard medical imaging that extracts a large number of quantitative features from CT, MRI, and PET images through advanced image analysis tools combined with statistical analysis and is now widely used in various clinical fields, such as increasing precision in diagnosis, predicting prognosis and therapy response (Hassani et al., 2019; Chu et al., 2021; Liang et al., 2021; Zhai et al., 2021; Guiot et al., 2022).

Recently, some studies have used a single MRI sequence or ADC values to construct prediction models for the prediction of MB molecular subtypes (Iv et al., 2019; Gonçalves et al., 2022; Saju et al., 2022). However, the results are still unstable, and there are fewer reports on constructing prediction models based on multiparametric MRI combined with clinical parameters for the prediction of MB molecular subtype. It was found that SHH and G4 were the most common molecular subtypes of MB in children, and the prognosis differed significantly between these two subtypes (Taylor et al., 2012). Total resection of the tumor in patients with G4 type has great importance in improving progression-free survival, especially in the presence of metastatic tumor spread preoperatively (Packer and Vezina, 2008; Thompson et al., 2016, 2018). Therefore, early preoperative prediction of molecular subtypes can help tailor individualized treatment and improve long-term prognosis.

In this study, we retrospectively analyzed MRI images and clinical data of 95 patients with MB to construct a machine learning model based on radiomics of multiparametric MRI combined with clinical parameters for predicting SHH and G4 molecular subtypes of pediatric MB.

## 2. Materials and methods

### 2.1. Patient characteristics

The Institutional Review Committees of our hospital approved the study. MRI imaging data and clinical data of patients with MB who were treated and followed up in the Children’s Hospital of Chongqing Medical University and the Children’s Hospital of Fudan University from October 2015 to October 2022 were collected. Inclusion criteria were: (1) availability of sufficient image quality preoperative, including axial T1, T2, T1C, T2FLAIR, and ADC maps; (2) availability of postoperative pathological and molecular subtype; (3) availability of complete clinical follow-up data. Exclusion criteria: molecular subtypes that could not be modeled due to the small number of cases were excluded. Exclusion criteria were: molecular subtypes that have fewer cases failed to build a model. 12 cases of WNT, 17 cases of G3, and 1 case of not otherwise specified (NOS) were excluded. Finally, 47 cases of SHH group and 48 cases of G4 group (90 cases from the Children’s Hospital of Chongqing Medical University and 5 cases from the Children’s Hospital of Fudan University) were enrolled.

### 2.2. Detection methods of molecular subtypes

The acquisition of molecular subtypes includes transcriptome-related assays and genome-related assays. Transcriptome assay use RNAseq methods to detect the expression levels of genes in the subjects, which can assist in determining the molecular subtype and prognosis of MB through the assessment of the expression levels of genes related to molecular typing. Genomic assay cover common variant types, including point mutations, insertions, deletions, amplifications, and fusions, in 687 genes related to tumors. This includes genetic variants highly related to the molecular subtype of MB molecular typing and other genetic variants related to molecular typing and drug use.

### 2.3. MRI acquisition

All patients underwent brain MR imaging at 1.5T or 3.0T (Signa EXCITE HD, GE Healthcare, Chicago, IL, United States; Discovery MR750, GE Healthcare, Milwaukee, WI, United States; Achieva, Philips Healthcare, Best, Netherlands), with scanning sequences encompassing axial T1, T2, T2FLAIR, T1c, and DWI (*b*-value taken as 1,000, with subsequent post-processing for ADC map generation). Details of the parameters for all the sequence acquisition are available in Supplementary Table 1.

### 2.4. Image uploading and tumor sketching

**FIGURE 1:**
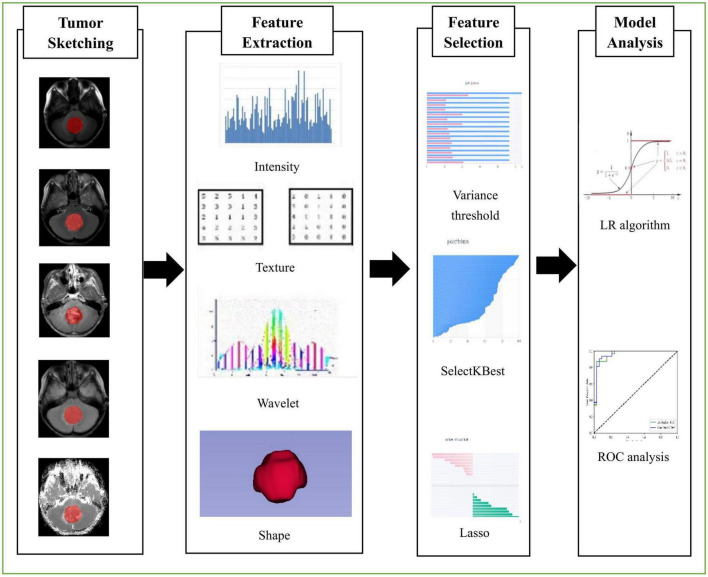
Flowchart shows the process of radiomics in this study, including tumor sketching, feature extraction, feature selection, and model analysis.

The steps of radiomics analysis are shown in Figure 1. Five sequence images of each patient were uploaded using the big data artificial intelligence research cloud platform developed by Huiying Medical Technology (Beijing) Co. A physician manually outlined regions of interest (ROI) for each sequence, layer by layer, for each case, using the platform’s built-in tools. An automatic computer-generated 3D volume of interest (VOI) of the lesion was obtained. The tumor boundary was outlined without peritumoral edema and reviewed by an experienced pediatric radiologist with 10 years of expertise in neuroimaging. If the regional variation was more than 5%, the boundary was decided by a senior physician, and neither of the two physicians knew the patient’s information during this procedure.

### 2.5. Radiomics feature extraction

A total of 7,045 quantitative imaging radiomics features were extracted from the outlined ROIs using the open source Python language environment toolkit Pyradiomics based on the Huiying Big Data research platform, and these features can be classified into four categories: ➀ Intensity statistics characterization: The distribution of voxel intensities within MR images is described quantitatively by commonly used and basic metrics. ➁ Shape and size features: These features reflect the shape and size of the ROI. ➂ Texture features: Based on the gray level run-length and gray level co-occurrence texture matrix calculation, we get the texture features that can quantify the difference of regional heterogeneity. ➃ High-order statistical features, intensity, and texture features of the transformed image are calculated again using various filters such as exponential, logarithmic, square, square root, and wavelet (including wavelet-LHL, wavelet-LHH, wavelet-HLL, wavelet-LLH, wavelet-HLH, wavelet-HHL, and wavelet-LLL).

### 2.6. Feature selection

As described above, we extracted quantitative imaging radiomics features from the five-sequence image ROIs of 95 patients. However, it is unlikely that all of these extracted features will be useful for a given task. Therefore, using feature downscaling to filter the specific features that are most relevant to this study for best performance is a necessary step. To reduce redundant features, feature selection methods include variance threshold (threshold value = 0.8), SelectKBest, and the least absolute shrinkage and selection operator (LASSO). For the variance threshold, the threshold value is 0.8, so feature values with variances less than 0.8 are removed. The SelectKBest method is a univariate feature selection method that uses *p*-values to analyze the relationship between features and classification results, which will allow screening all features with *p*-values less than 0.05. For LASSO regression, the L1 regularizer is used as the cost function, with a maximum number of iterations of 1,000. Finally, we obtain 17 optimal feature subsets.

### 2.7. Machine learning classification

Based on the selected feature subsets, the LR algorithm was used to construct the radiomics feature model and the combined model of radiomics features and clinical parameters, respectively, and the patients were divided into training and testing cohorts by the random grouping method in the ratio of 7:3 to obtain the classification prediction results.

### 2.8. Statistical analysis

Clinical data were statistically analyzed using SPSS 25.0 statistical software. Measurement data that conforms to a normal distribution were presented as *x* ± *s*, and the independent samples *t*-test was used to compare the differences between groups. Categorical data were tested using the χ^2^ test or Fisher’s exact test, and differences were considered statistically significant at *P* < 0.05. For the machine learning results, ROC curves were used in the training cohort and testing cohort to compare model prediction accuracy and calculate AUC, sensitivity, and specificity. The Delong test was used to compare the AUC differences between the ROC curves of the two models, and *P* < 0.05 was considered statistically significant.

## 3. Results

### 3.1. Clinical features

A total of 95 patients with MB were enrolled in this study, including 47 cases of SHH and 48 cases of G4. The clinical data such as gender, age, tumor location, clinical symptoms and physical signs, hydrocephalus status, metastasis or recurrence status, blood biochemical indexes, and pathological typing were collected. There were no significant differences in age, gender, renal function, liver function, intracranial hypertension, ataxia and preoperative metastases, recurrence or metastases at follow-up between these two groups (*P* > 0.05). Pre-dominant pathological typing was desmoplastic or nodular in SHH and classic in G4. The tumor location was mainly in the cerebellum (cerebellar hemisphere or pontine arm) in SHH and in the midline (four ventricles or cerebellar vermis) in G4. Compared with patients in SHH, those in G4 had a higher likelihood of developing hydrocephalus. These differences between groups were statistically significant (*P* < 0.05), as shown in Table 1.

**TABLE 1:** Clinical information of SHH and G4 groups.

Clinical data	SHH (*n* = 47)	G4 (*n* = 48)	*P*-value
**Hydrocephalus**			
Absent	35	45	0.022
Present	12	3	
**Location**			
Cerebellum	23	2	0
Midline	24	46	
**Pathological typing**			
Classic	11	46	0
Desmoplastic or nodular	32	0	
Large cell or anaplastic	2	2	
Extensive nodularity	2	0	
**Age (*x* ± *s*, year)**	6.75 ± 3.54	7.50 ± 2.97	0.217
**Sex**			
Female	15	18	0.568
Male	32	30	
**Renal function**			
Abnormal	1	0	0.495
Normal	46	48	
**Liver function**			
Abnormal	2	1	0.985
Normal	45	47	
**Preoperative metastases**			
Absent	7	6	0.734
Present	40	42	
**Intracranial hypertension**			
Absent	40	45	0.299
Present	7	3	
**Ataxia**			
Absent	13	18	0.306
Present	34	30	
**Follow up recurrence or metastases**			
Absent	12	9	0.426
Present	35	39	

### 3.2. Radiomic features

A total of 7,045 quantitative imaging features were extracted from the five sequence images of 95 patients, and 17 optimal feature sets, including 13 texture features and 4 intensity features, were obtained after dimensionality reduction using variance threshold (threshold = 0.8), SelectKBest, and the LASSO regression algorithm. The details were shown in Table 2.

**TABLE 2:** Seventeen optimal radiomic features.

Radiomic feature	Radiomic class	Filter
High gray level zone emphasis is	glszm	MB-ADC_wavelet-HHL
High gray level zone emphasis	glszm	MB-FLAIR_wavelet-LHH
Large dependence low gray level emphasis	gldm	MB-T2_wavelet-LLL
Run variance	glrlm	MB-FLAIR_wavelet-LLH
Busyness	ngtdm	MB-ADC_wavelet-LLH
Zone entropy	glszm	MB-FLAIR_wavelet-LLH
Zone variance	glszm	MB-FLAIR_wavelet-HHL
Skewness	firstorder	MB-T1_wavelet-HLL
Kurtosis	firstorder	MB-ADC_exponential
Dependence variance	gldm	MB-FLAIR_wavelet-LHL
High gray level zone emphasis	glszm	MB-T1C_wavelet-HHL
Size zone non-uniformity	glszm	MB-FLAIR_wavelet-HLL
Variance	firstorder	MB-T1C_wavelet-LLL
Small area high gray level emphasis	glszm	MB-ADC_wavelet-HHH
Zone entropy	glszm	MB-FLAIR_original
Zone entropy	glszm	MB-FLAIR_logarithm
Range	firstorder	MB-FLAIR_logarithm

### 3.3. Model performance

**FIGURE 2:**
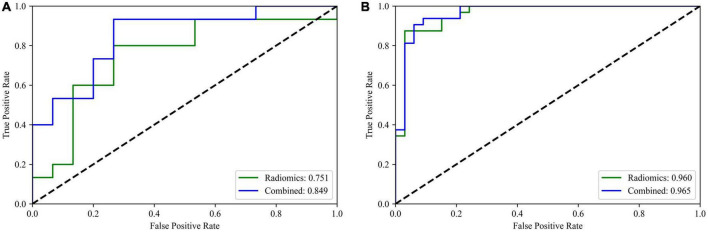
**(A,B)** ROC curves of the radiomics and combined model on the testing cohort and training cohort, respectively.

**FIGURE 3:**
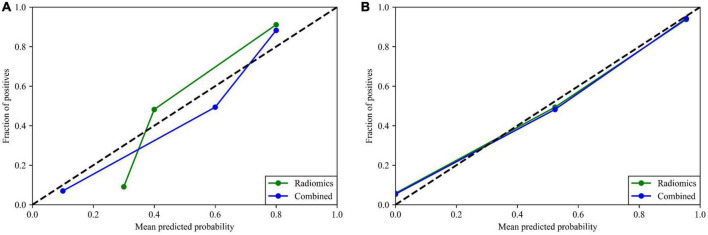
**(A,B)** Calibration curves of the radiomics and combined model on the testing cohort and training cohort, respectively.

**FIGURE 4:**
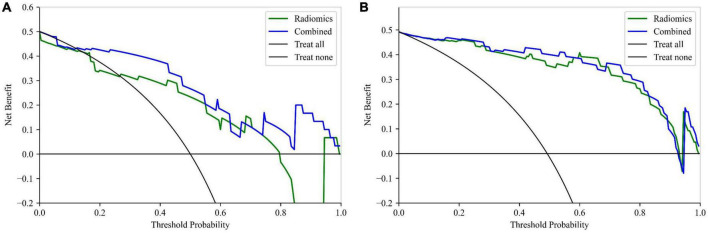
**(A,B)** Decision curves of the radiomics and combined model on the testing cohort and training cohort, respectively.

**FIGURE 5:**
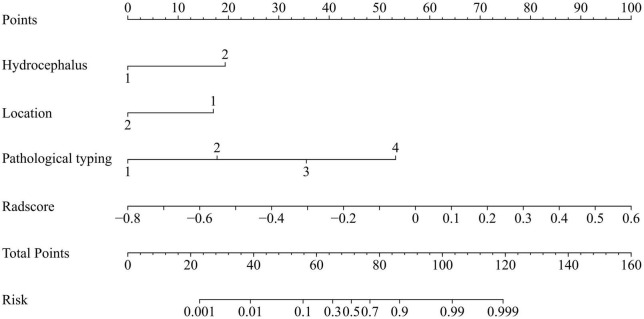
Nomogram based on clinical and radiomics score.

In the radiomics feature model, the AUC was 0.960 (95% CI: 0.871−1.000), and the sensitivity and specificity were 0.880 and 0.850, respectively, in the training cohort. In the testing cohort, the AUC was 0.751 (95% CI: 0.587−0.915), and the sensitivity and specificity were 0.730 and 0.730, respectively. In the radiomics features and clinical parameters combined model, the AUC was 0.965 (95% CI: 0.898−1.000), and the sensitivity and specificity were 0.910 and 0.940, respectively, in the training cohort. In the testing cohort, the AUC was 0.849 (95% CI: 0.695−1.000), and the sensitivity and specificity were 0.800 and 0.730, respectively. The ROC curves for both the training and testing cohorts are shown in Figures 2A, B and Tables 3, 4. The calibration curves show the goodness of fit between the predicted molecular subtypes and actual molecular subtypes in both the training and testing cohort for the radiomics model and the combined model (Figures 3A, B). The decision curves show that the combined model outperforms the radiomics model in terms of net benefit (Figures 4A, B). The clinical utility of both prediction models is demonstrated by the nomogram (Figure 5).

**TABLE 3:** AUC, 95% CI, sensitivity, and specificity of radiomics model in training cohort and testing cohort.

Cohort	AUC	95% CI	Sensitivity	Specificity
Training cohort	0.960	0.871−1.000	0.880	0.850
Testing cohort	0.751	0.587−0.915	0.730	0.730

**TABLE 4:** AUC, 95% CI, sensitivity and specificity of combined model in training cohort and testing cohort.

Cohort	AUC	95% CI	Sensitivity	Specificity
Training cohort	0.965	0.898−1.000	0.910	0.940
Testing cohort	0.849	0.695−0.915	0.800	0.730

Delong test results showed the difference of prediction accuracy measured by AUC in the testing cohort of the two prediction models has statistical significance. The results were shown in Table 5.

**TABLE 5:** Delong test result.

	Training cohort radiomics model/Combined model	Testing cohort radiomics model/Combined model
AUC	0.960/0.965	0.751/0.849
*P*-value	0.4604	0.0144

## 4. Discussion

Medulloblastoma is a highly aggressive brain tumor, the therapeutic strategies and clinical prognosis vary significantly among molecular subtypes. The published methods of molecular classification are invasive, relying mostly on gene expression and methylation analyses. Due to the tumor tissue heterogeneity, the biopsy tissue specimens cannot fully capture the whole tumor tissue information. In this study, we extracted radiomics features of the entire tumor region and combined them with clinical parameters to build a prediction model for SHH and G4 molecular subtypes of pediatric MB. The results showed that the combined model had significant classification efficacy with an AUC > 0.8 in the testing cohort, potentially providing a non-invasive clinical approach to preoperatively predict SHH and G4 molecular subtypes of MB.

In this study, we used clinical characteristics, including hydrocephalus status, tumor location, and pathological type, to construct a combined model for predicting SHH and G4 molecular subtypes of MB. In our study, there were no significant differences in age and gender between the two molecular subtypes, which is not consistent with the previous studies reported by Taylor et al. (2012) and Eid and Heabah (2021). We suspect that this may be due to the small sample size of our cohort. Similar to a recent study by Yan et al. (2020) that enrolled 122 patients, our study also showed that the tumor location and hydrocephalus status differed in molecular subtypes and were combined in the model to improve predictive efficacy.

In our study, 17 optimal radiomics features were selected, containing 13 texture features and 4 intensity features. A multicenter study of 263 patients from 12 children’s hospitals reported that texture features and first-order intensity features contributed the most to improving the predictive efficacy of the model (Zhang et al., 2022). In the study by Yan et al. (2020), three texture features and eight intensity features were extracted to construct the model. Also, in the study by Chang et al. (2021), 38 children from Taipei showed significant differences in eight textural features among different molecular subtypes. In a systematic review and meta-analysis on radiomics-based machine learning for predicting molecular subtypes of MB (Karabacak et al., 2022), five articles enrolled 420 patients with MB, and the results showed that the mean AUC of prediction models for all MB molecular subtypes was >0.8, indicating a greater possibility of predicting MB molecular subtypes by radiomics studies. Compared with the above-mentioned studies, our study used comprehensive image features of five sequences including T1, T2, T1C, T2FLAIR, and ADC maps, and combined them with clinical parameters to construct a combined model with improved prediction accuracy. The AUC was 0.965 in the training cohort and 0.849 in the testing cohort, respectively, which can be used to facilitate the prediction of SHH and G4 molecular subtypes of MB preoperatively.

This study had some limitations. First, the number of cases in the SHH and G4 groups enrolled in this study was relatively small, and the WNT and G3 groups were not included in the study because the number of cases did not meet the modeling requirements. Further expansion of the sample size is needed to conduct a multicenter, prospective study. Second, the results of this study lack external validation to better assess the generalizability of the model, which still needs further investigation.

## 5. Conclusion

In summary, our study demonstrated that a combined prediction model based on the radiomics features of multiparametric MRI and clinical parameters can effectively predict the SHH and G4 molecular subtypes of MB prior to surgery. These findings highlight the potential of radiomics and machine learning techniques for non-invasive preoperative prediction of MB molecular subtypes.

## Data availability statement

The original contributions presented in this study are included in the article/Supplementary material, further inquiries can be directed to the corresponding authors.

## Ethics statement

Written informed consent was obtained from the individual(s), and minor(s)’ legal guardian/next of kin, for the publication of any potentially identifiable images or data included in this article.

## Author contributions

YW wrote the manuscript and which was revised by the YZ and LL. LW, BQ, and YJ contributed to the data processing and statistical analysis. WX, XH, ZT, and SL contributed to the data collection. All authors contributed to the article and approved the submitted version.
