# Supplementary material for: Preoperative prediction of sonic hedgehog and group 4 molecular subtypes of pediatric medulloblastoma based on radiomics of multiparametric MRI combined with clinical parameters
Source: Front Neurosci. 2023 Apr 11;17:1157858. doi: 10.3389/fnins.2023.1157858 (PMC10126354; doi:10.3389/fnins.2023.1157858)
Supplement: Supplementary file 1 [file Table_1.DOCX]

| Sequence |  | GE  1.5T | GE  3.0T | Philips  3.0T |
| --- | --- | --- | --- | --- |
| T1WI | TR (ms) | 2000-3000 | 2000-3000 | 2000-3000 |
|  | TE (ms) | 7-40 | 20-40 | 20-40 |
|  | TI (ms) | 800-1000 | 800-1000 | 800-1000 |
|  | Slice thickness (mm) | 6.0-6.5 | 6.0-6.5 | 6.0-6.5 |
| T2WI | TR (ms) | 2200-5000 | 2200-6000 | 2200-6000 |
|  | TE (ms) | 80-110 | 80-110 | 80-110 |
|  | Slice thickness (mm) | 6.0-6.5 | 6.0-6.5 | 6.0-6.5 |
| T2FLAIR | TR (ms) | 8000 | 8000 | 8000 |
|  | TE (ms) | 120-150 | 120-130 | 120-130 |
|  | TI (ms) | 2100-2500 | 2100-2400 | 2100-2500 |
|  | Slice thickness (mm) | 6.0-6.5 | 6.0-6.5 | 6.0-6.5 |

**Supplementary Table 1:** MRI scanners and parameters.
